# Validation of a mechanism to balance exercise difficulty in robot-assisted upper-extremity rehabilitation after stroke

**DOI:** 10.1186/1743-0003-9-6

**Published:** 2012-02-03

**Authors:** Lukas Zimmerli, Carmen Krewer, Roger Gassert, Friedemann Müller, Robert Riener, Lars Lünenburger

**Affiliations:** 1 Sensory-Motor Systems Lab, Department of Mechanical Engineering and Process Engineering, ETH Zurich, Zurich, Switzerland; 2 Hocoma AG, Volketswil, Switzerland; 3 Schoen Klinik Bad Aibling, Bad Aibling, Germany; 4 Rehabilitation Engineering Lab, Department of Mechanical Engineering and Process Engineering, ETH Zurich, Zurich, Switzerland; 5 Medical Faculty, Balgrist University Hospital, University of Zurich, Zurich, Switzerland

## Abstract

**Background:**

The motivation of patients during robot-assisted rehabilitation after neurological disorders that lead to impairments of motor functions is of great importance. Due to the increasing number of patients, increasing medical costs and limited therapeutic resources, clinicians in the future may want patients to practice their movements at home or with reduced supervision during their stay in the clinic. Since people only engage in an activity and are motivated to practice if the outcome matches the effort at which they perform, an augmented feedback application for rehabilitation should take the cognitive and physical deficits of patients into account and incorporate a mechanism that is capable of balancing i.e. adjusting the difficulty of an exercise in an augmented feedback application to the patient's capabilities.

**Methods:**

We propose a computational mechanism based on Fitts' Law that balances i.e. adjusts the difficulty of an exercise for upper-extremity rehabilitation. The proposed mechanism was implemented into an augmented feedback application consisting of three difficulty conditions (easy, balanced, hard). The task of the exercise was to reach random targets on the screen from a starting point within a specified time window. The available time was decreased with increasing condition difficulty. Ten subacute stroke patients were recruited to validate the mechanism through a study. Cognitive and motor functions of patients were assessed using the upper extremity section of the Fugl-Meyer Assessment, the modified Ashworth scale as well as the Addenbrookes cognitive examination-revised. Handedness of patients was obtained using the Edinburgh handedness inventory. Patients' performance during the execution of the exercises was measured twice, once for the paretic and once for the non-paretic arm. Results were compared using a two-way ANOVA. Post hoc analysis was performed using a Tukey HSD with a significance level of p < 0.05.

**Results:**

Results show that the mechanism was capable of balancing the difficulty of an exercise to the capabilities of the patients. Medians for both arms show a gradual decrease and significant difference of the number of successful trials with increasing condition difficulty (*F_2;60 _*= 44.623; *p *< 0.01; *η^2 ^*= 0.623) but no significant difference between paretic and non-paretic arm (*F_1;60 _*= 3.768; *p *= 0.057; *η^2 ^*= 0.065). Post hoc analysis revealed that, for both arms, the hard condition significantly differed from the easy condition (*p *< 0.01). In the non-paretic arm there was an additional significant difference between the balanced and the hard condition (*p *< 0.01). Reducing the time to reach the target, i.e., increasing the difficulty level, additionally revealed significant differences between conditions for movement speeds (*F_2;59 _*= 6.013; *p *< 0.01; *η^2 ^*= 0.185), without significant differences for hand-closing time (*F_2;59 _*= 2.620; *p *= 0.082; *η^2 ^*= 0.09), reaction time (*F_2;59 _*= 0.978; *p *= 0.383; *η^2 ^*= 0.036) and hand-path ratio (*F_2;59 _*= 0.054; *p *= 0.947; *η^2 ^*= 0.002). The evaluation of a questionnaire further supported the assumption that perceived performance declined with increased effort and increased exercise difficulty leads to frustration.

**Conclusions:**

Our results support that Fitts' Law indeed constitutes a powerful mechanism for task difficulty adaptation and can be incorporated into exercises for upper-extremity rehabilitation.

## Background

Motor skills are indispensable to interact with and navigate through our social environment. Neurological disorders such as spinal cord lesions, stroke and traumatic brain injuries frequently affect these motor functions. Since these impairments influence a person's participation in social and economic life, their restoration through rehabilitation plays an important role in improving patients' quality of life [1].

In recent years the use of robotic interventions have become more and more popular in the field of rehabilitation. Upper- and lower-extremity devices allow patients to participate more actively during therapy, allow longer training periods and more precise repetitions of the motor patterns that have to be regained and assist and reduce the workload of therapists during rehabilitation on a daily basis [2-9]. Nevertheless, patients still rely on physiotherapists to keep them motivated and engaged during therapy and give them feedback about the quality of their movements.

With the advent of modern media technologies, the use of augmented feedback has nowadays become more and more prominent. Augmented feedback applications use digital displays e.g. computer screens and virtual reality (VR) [10,11] to provide patients with an external feedback source about their motor performance. If correctly applied, augmented feedback applications allow precise movement feedback, supplementing proprioception, and through game-like scenarios increase the overall motivation and engagement during training-key ingredients for productive motor learning and an important factor for successful rehabilitation [1,12,13].

Many augmented feedback applications have been developed in the past years. They can generally be divided into applications that increase motivation through improved graphics and challenging exercises as well as applications that concentrate more on direct feedback and lack the motivating aspects of the former [9,14-24]. Most of these applications are, however, only capable of training a certain aspect of the movement and include a set of predefined exercise difficulty levels. Thus the therapist faces the challenge of deciding on the right application and exercise difficulty that is suitable and most effective for the patient's current stage of recovery.

Due to the increasing number of patients, increasing medical costs and limited therapeutic resources, clinicians may in the future want patients to practice their movements at home or with reduced supervision during their stay in the clinic. The need for augmented feedback applications that are capable of keeping a patient engaged and motivated while respecting a patients' abilities will hence be desirable. Studies have shown that people only engage in an activity if the outcome matches the effort at which they perform [25-27]. Hence learners who believe that they are competent or successful have been shown to remain engaged and motivated over a longer period of time [28]. In our view, this can only be achieved if an augmented feedback application considers the cognitive and physical deficits of patients and incorporates a mechanism that is capable of balancing the difficulty of an exercise i.e. adapt the difficulty to the current capabilities of the patient.

Several studies have already addressed the need for balancing mechanisms in augmented feedback applications for upper-extremity rehabilitation [17,29,30] as well as in specifically developed robotic devices [31,32]. While the proposed approaches in augmented feedback applications have shown to be functional they require the evaluation of the effects that different properties of VR elements have on the level of difficulty, e.g., object size or speed of exercise elements. This can be a complex and enduring process that has to be repeated for each particular application and hence complicates the adaptation to different exercises. Therefore, we propose a different mechanism for upper-extremity rehabilitation that we believe is capable of adapting the difficulty of an exercise. It founds upon a well established empirical formula, Fitts' Law [33], that natively already incorporates and describes different parameters and their effects on the level of difficulty. Since Fitts' Law is a physiologically valid and widely used descriptor of reaching motions it can be applied to a variety of different upper-extremity exercises. It has successfully been shown that Fitts' Law, which originated from one-dimensional, real-world observations can also be applied to computer screens and input devices [34-36] and is valid in the two dimensional space [37]. The goal of this paper was to apply this mechanism to an augmented feedback application and verify its applicability and validity through a study with ten stroke patients. Since Fitts' law is capable of describing reaching motions, we hypothesize that its application as a balancing mechanism will result in exercise difficulties that are adapted to patients capabilities. We believe that this will lead to a challenging instead of a frustrating therapy experience.

### Fitts' Law

Fitts' Law (Equation 1) describes a linear relationship between the time (T) needed to move from a start to a target location and the properties these locations possess i.e. the size of the target (W) and the distance between them (D). The logarithmic term of this relationship has been termed "Index of Difficulty" (ID). Equation 1 shows the Shannon formulation of Fitts' Law, which has been shown to fit measured data of low IDs better compared to the original formulation [35]. The relationship between movement time and ID can be obtained by measuring movement times for a number of different IDs and performing a linear regression on the acquired data. This will yield values for intercept (a) and slope (b) which are both distinctive for a person's or patient's current reaching motion and the input device that was used.

(1) $$T=a+b\;\log_{2}\left(1+\frac{D}{W}\right)$$

Since upper-extremity rehabilitation of stroke patients is concerned with the reacquisition of reaching motions, Fitts' Law allows to assess the current quality of a patient's movement, thus yielding information about his/her capabilities [38-40]. This information could hence also be used by a mechanism to adjust the difficulty of an exercise.

## Methods

### Subjects

Ten patients with hemiparesis in the subacute phase were enrolled in the study at the Schoen Klinik Bad Aibling, Germany. All met the following inclusion criteria: age between 18 and 75 years (*mean age: 51.22 +- 18.30*); hemiparesis after first-time, unilateral stroke; time between lesion and enrollment into the study between 3 weeks and 6 months. Exclusion criteria were: other neurological disorders (e.g. Parkinson's disease, diabetic polyneuropathy), severe aphasia or dementia (not able to understand the informed consent). In order to obtain an overview of the cognitive and motor functions of the subjects, their physical skills were assessed using the upper extremity section of the Fugl-Meyer Assessment (FMA; *mean: 39.40 +- 10.71*) [41] and the modified Ashworth scale [42] while their cognitive functions were measured using the Addenbrookes cognitive examination-revised (ACE-R; *mean: 78.44 +-15.15*), with a focus on visual-spatial deficit perception (ACE-R visuo-spatial subscore; mean: 14.2 +-2.1) [43]. To identify the handedness of patients in daily activities on a quantitative scale, the Edinburgh handedness inventory (EHI) was employed [44] (see Table 1). Approval was obtained from the Bavarian State Board of Physicians. All patients or their legal representatives gave their written informed consent before participating in the study.

**Table 1:** List of patients that participated in the study

Patient [#]	Sex	Age [y]	Time since stroke [months]	Paretic Arm	Lesion Location/Type	FMA	Ashworth	ACE-R (visuo-spatial subscore)	EHI
1	m	55.8	3.7	left	MCA/Ischemic	43	1;0;2;1;0;0	52 (10)	right
2	m	68.3	2.5	left	watershed stroke/Ischemic	48	0;0;0;0;0;0	-	right
3	m	37.2	4.3	right	MCA/Ischemic	48	0;0;1+;0;1;0	89 (16)	right
4	f	73.1	2.7	left	Basal Ganglia/Hemorrhagic	37	0;1;0;1;0;0	64 (13)	right
5	m	38.6	1.6	left	MCA/Ischemic	54	0;0;0;0;0;0	74 (15)	right
6	m	56.8	2.5	right	Basal Ganglia/Hemorrhagic	26	0;0;1+;0;1+;0	67 (12)	right
7	f	69.8	2.0	left	MCA/Ischemic	31	0;1;0;1;1;0	91(16)	right
8	m	23.8	2.0	right	MCA/Ischemic	30	0;0;1;0;2;0	84 (16)	left
9	m	26.4	1.6	left	MCA, ACA/Ischemic	51	1;1;1;1;1+;0	92 (15)	right
10	m	62.4	2.0	right	Pons/Ischemic	26	0;0;1;0;+1;0	94 (15)	right

m: male, f: female, MCA: Middle cerebral artery, ACA: Anterior cerebral artery, FMA: Upper-Extremity section of the Fugl-Meyer Assessment, Ashworth: modified Ashworth Scale (Order: shoulder flexors, shoulder extensors, elbow flexors, elbow extensors, wrist flexors, wrist extensors), ACE-R: Addenbrookes cognitive examination-revised, EHI: Edinburgh handedness inventory

### Weight compensation system

**Figure 1:**
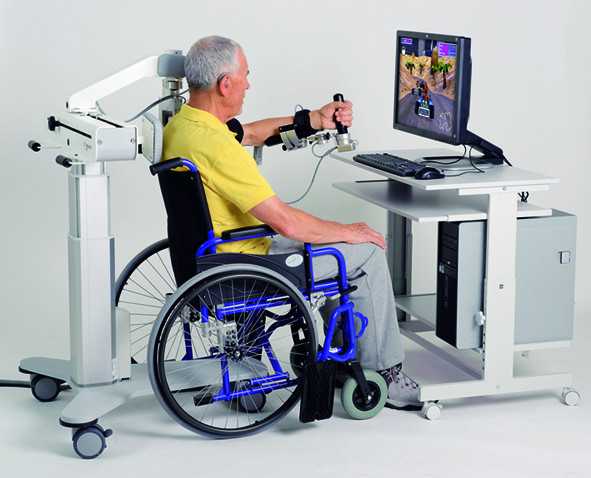
**The experimental setup**. Experimental setup using a passive device for upper-extremity rehabilitation (Armeo^®^Spring, Image courtesy of Hocoma AG, Switzerland).

The study was performed using a passive 5 degrees of freedom weight compensation system (Armeo^®^Spring, Hocoma AG, Switzerland, see Figure 1, commercial version of T-WREX [4,6]). Through integrated springs, the orthosis counterbalances the patient's paretic arm against gravity. This enhances any residual functions of the patient, enabling the training of active reaching movements. At the tip of the exoskeleton, the device includes a pressure sensitive handgrip that can trace hand grip force. Electronic sensors that measure movement and grip force allow patients to interact with augmented feedback applications (see Figure 1). In addition to physically supporting the patient's arm, the device further measures the range of motion of each patient to allow full interactions with the virtual workspace.

### Augmented Feedback Application

**Figure 2:**
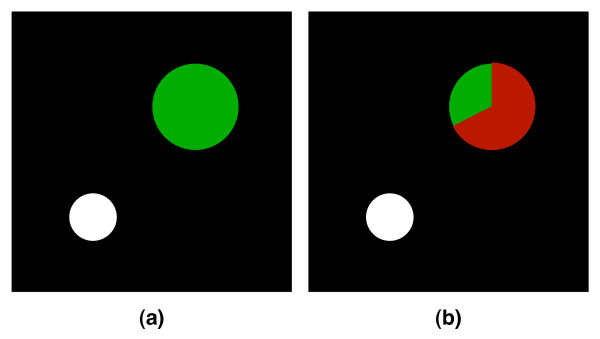
**The augmented feedback application**. (a) and (b) illustrate the two phases of the augmented feedback application using Fitts' Law. Unlike during the "assessment phase" (a), patients received additional information about the time left to reach the target during the "exercise phase" (b) through a change of color of the target location. White circle: Start location, Green circle: Target location, Red-Area: Time indicator.

The application we developed to evaluate the balancing mechanism can be divided into two phases. In the first phase, the "assessment phase", Fitts' Law was used to assess the patient's performance. The slope and intercept that were gained through this assessment were used in the second phase, the "exercise phase", to estimate the time that a patient would need to move between randomly chosen start and target locations of different sizes, i.e., different IDs. Assessment and exercise phase were both performed using the same augmented feedback application and task. The only difference was that while there was a time restriction during the exercise phase, this was not the case during the assessment phase. In order to interact with the augmented feedback applications, real world movements performed parallel to the frontal plane were mapped onto the mouse position on the screen, giving patients continuous spatial position feedback. Each movement from start to target location was defined as a trial, and can be subdivided into three different parts. The "initiation part" i.e. the starting of the movement, the "movement part" i.e. the movement between start and target location, and the "closure part" i.e. the trial completion that patients performed by closing their hand when they reached the target. In order to correctly initiate the timer that measured the movement time, patients were instructed to wait one second over the starting position before moving towards the target location. The movement initiation trigger was given by a change in color of the target location from light to dark green accompanied by an auditory cue. Unlike during the "assessment phase", where no timing information was available, patients received a visual feedback during the "exercise phase" indicating the time left to reach the target through a gradual change in color of the target location from green to red. A positive auditory cue was played if the trial was completed on time, indicating a successful trial, while a negative auditory cue was presented if the target was reached too late (see Figure 2), indicating an unsuccessful trial.

### Study-Design

**Figure 3:**
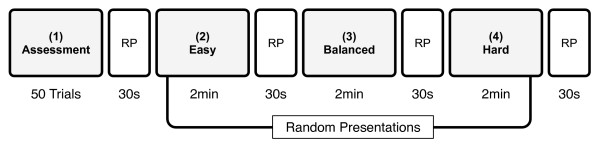
**Study Design**. The study was composed of an assessment (1), an easy (2), balanced (3) and hard (4) condition. In the easy condition, the patient had twice the amount of the estimated time for the movement. In the balanced condition the available time for the movement was equal to the estimated time and in the hard condition the patient had half the estimated time for the movement. Each condition was followed by a rest period (RP) during which the NASA-TLX questionnaire was deployed to measure subjective feeling of condition difficulty. While the assessment condition was always presented at the beginning of the experiment and comprised 50 trials, conditions 2-4 were randomly presented for 2 min each.

The study design comprised four different conditions (see Figure 3): (1) an assessment phase, which was used to measure 50 different movement times (5 IDs × 10 trials) and calculate the intercept and slope of Fitts' Law. Since intercepts and slopes were needed by the balancing mechanism to estimate the movement times, this condition was always at the beginning of an experiment. After the assessment, three conditions with different difficulty levels were randomly presented to the patients. In the easy condition (2) the patient was given double the estimated movement time to reach the target, in the balanced condition (3) the available time was equal to the estimated time and in the hard condition (4) patients had half the time to complete the movement. The easy and the hard conditions were added to the study design in order to evaluate the balancing mechanism. This allowed direct comparisons between the performance during the different conditions and motivational aspects of the balanced condition to those of the easy and hard conditions. Easy and hard conditions were similarly based on the results of the "assessment phase" in order to make them relative to the capabilities of each patient. We believe that this resulted in more objective outcomes than if they would have been preset and equal for each subject. While there were a fixed number of trials that were measured during condition 1, conditions 2-4 were each presented for 2 min. To allow for rest between the different conditions, a 30 s rest period (RP) followed each condition. In order to obtain a better understanding of the balancing mechanism and to verify its applicability, the experiment was carried out twice per patient, once for the paretic and once for the non-paretic arm.

### Movement Measurements

**Figure 4:**
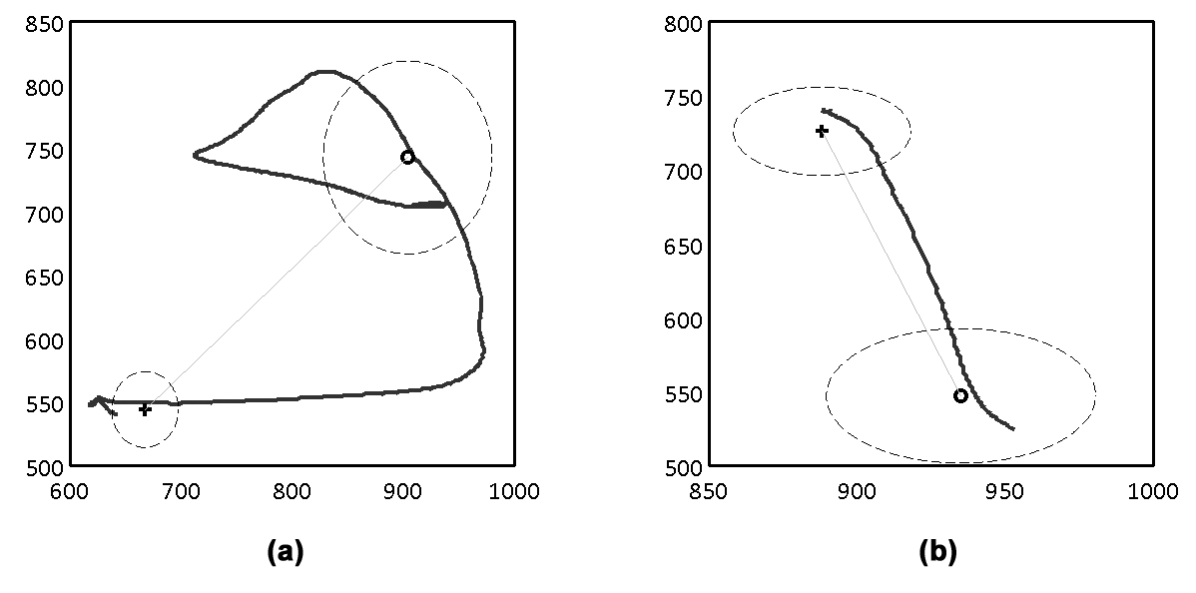
**Movement Trajectories**. (a) and (b) illustrate movement trajectories for the paretic and non-paretic arm of the same subject, respectively. One can clearly see the difference to the movements of the paretic arm being imprecise and uncontrolled compared to the ones of the non-paretic arm.

Subdividing each trial into different parts allowed measuring different functional aspects of reaching movements. Reaction time was measured by the lapse of time between the presentation of the movement cue and the patient leaving the starting location i.e. start circle. The "movement part" was used to measure movement trajectories in order to calculate hand-path ratios (HPR) and record the overall speed of the movement. HPR's are calculated by dividing the length of the movement trajectory by the most direct path between the start and target locations (Figure 4) e.g. a HPR of two would mean that the performed trajectory was twice as long as the straight line from start to target. Movement speed was obtained by dividing the distance between the start and target location by the total time of the "movement part". Eventually, the hand-closing time was measured during the "closure part" of the trial i.e. the elapsed time between the patient entering the target location an closing his hand.

### Questionnaire

During the RP, patients were asked to answer the six questions of the NASA Task Load Index (NASA-TLX; Table 2) [45,46] in order to assess their subjective measure of condition difficulty. Each question could be answered using scores from 0 (very low) up to 21 (very high).

**Table 2:** Questions of the NASA Task Load Index

Sub-Scales	Endpoints	Question
Mental Demand	Low/High	How mentally demanding was the task?
Physical Demand	Low/High	How physically demanding was the task
Temporal Demand	Low/High	How hurried or rushed was the pace of the task?
Performance	Good/Poor	How successful were you in accomplishing what you were asked to do?
Effort	Low/High	How hard did you have to work to accomplish your level of performance?
Frustration Level	Low/High	How insecure, discouraged, irritated, stressed and annoyed were you?

Used to obtain the subjective workload of a subject [45,46].

### Data Acquisition & Analysis

Experiments were conducted on a commercial PC with a screen resolution of 1280 × 1024 pixels using the weight compensation device as mouse input. All statistical analyses and plots were generated using IBM SPSS 19 (IBM Corporation, USA) and MATLAB R2009b (MathWorks, USA) on an Intel MacBook Pro. Parameters were compared using a two-way ANOVA. Post hoc analyses were performed using a Tukey HSD. The significance level was set to p < 0.05.

## Results

### Fitts' Law

To illustrate the values that were used for the estimation of the time needed for the movements during the easy, balanced and hard conditions, Table 3 shows the slopes, intercepts and correlation coefficients that were calculated based on data of the "assessment" condition. Correlation of the data is at R^2 ^> 0.7 for the paretic arm of patients 1, 2, 3, 9 and 10 and for the non-paretic arm of patients 4, 5, 6, 7, 8 and 10.

**Table 3:** List of the intercepts, slopes and correlations

	Paretic			Non-Paretic		
**Patient #**	**Intercept**	**Slope**	**R2**	**Intercept**	**Slope**	**R2**

1	5.2426	-3.8267	0.8498	0.749	-0.3296	0.4587
2	1.9382	-1.5806	0.713	0.897	0.7901	0.4967
3	1.5705	0.1127	0.7271	0.1922	0.5899	0.1951
4	2.5553	1.619	0.3558	17.0933	-20.7932	0.7202
5	-0.3324	3.3407	0.0231	0.5991	-0.0628	0.9003
6	0.0606	11.7751	0	2.1233	-1.5282	0.7625
7	2.4267	0.716	0.2298	6.6942	-6.0491	0.7944
8	3.0954	6.739	0.2199	0.3282	0.3761	0.7976
9	0.581	0.1949	0.7519	0.0979	0.5335	0.174
10	1.7999	-0.8733	0.8759	1.4094	-1.3197	0.7442

Obtained for the paretic and non-paretic arm using linear regression in the assessment condition.

### Successful Trials

**Figure 5:**
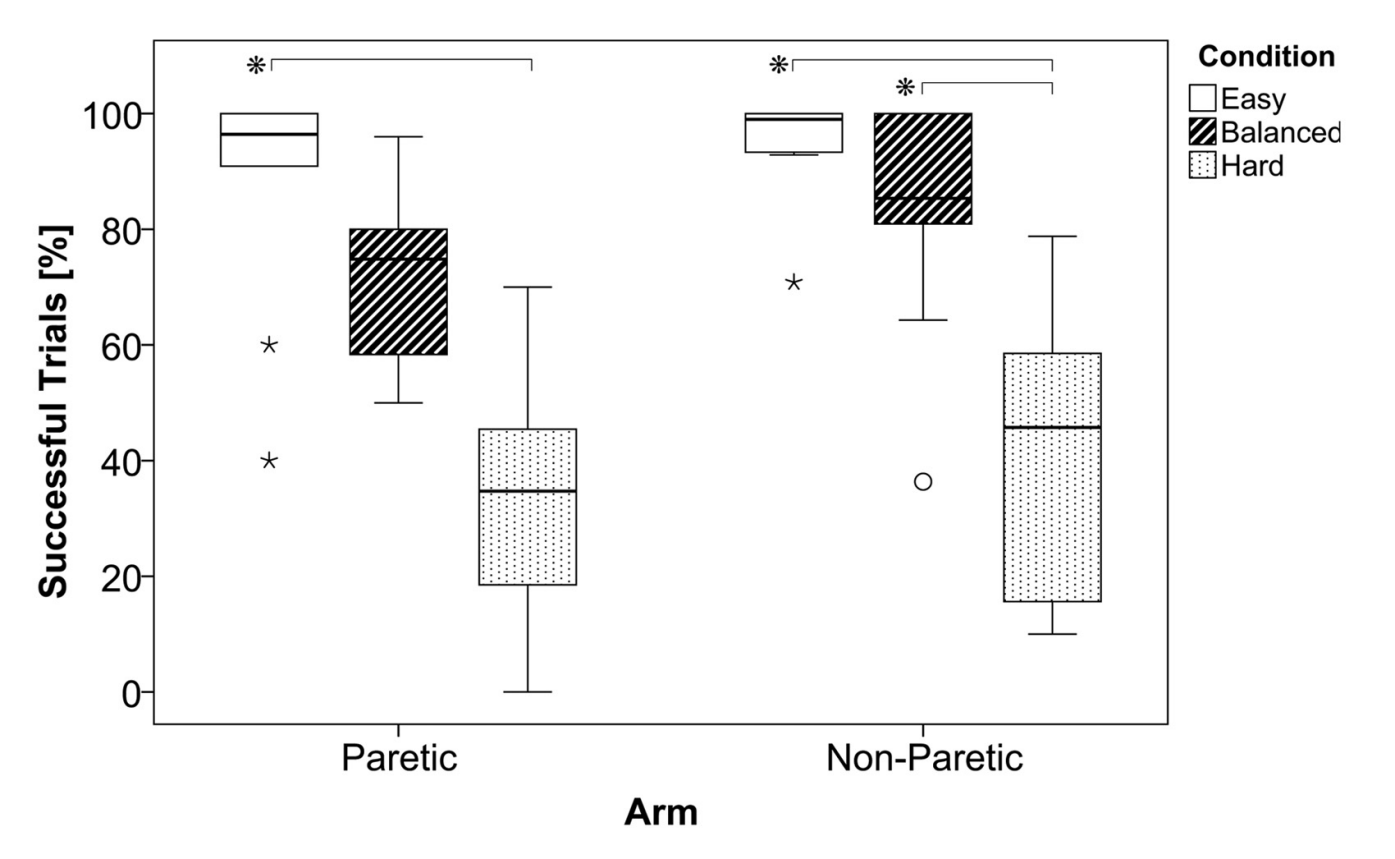
**Successful Trials**. Boxplots show the decreasing amount of successful trials over condition difficulty for the paretic and non-paretic arm. Bottom and top whiskers indicate the 25th and 75th percentile respectively. Whiskers extend to 1.5 times the height of the box or, to the minimum or maximum values if no case has a value in that range. Outliers (o) have values that do not fall within the whiskers and are marked with an asterisks if their values are more than three times the height of the boxes i.e. extreme outliers.

Results in Figure 5 show the percentage of successful trials that were achieved during the different conditions with the paretic and non-paretic arm. The medians for both arms show a gradual decrease of the number of successful trials with increasing condition difficulty. The ANOVA did not reveal a significant difference between the paretic and non-paretic arm (*F_1;60 _*= 3.768; *p *= 0.057; *η^2 ^*= 0.065), and showed a significant difference between the conditions (*F_2;60 _*= 44.623; *p *< 0.01; *η^2 ^*= 0.623), with no significant interactions between arm and conditions (*F_1;60 _*= 0.037; *p *= 0.964; *η^2 ^*= 0.001). Post hoc analysis revealed that, with both the paretic and the non-paretic arm, the hard condition significantly differed from the easy condition (*p *< 0.01). In the non-paretic arm there was an additional significant difference between the balanced and the hard condition (*p *< 0.01).

### Movement Speed

**Figure 6:**
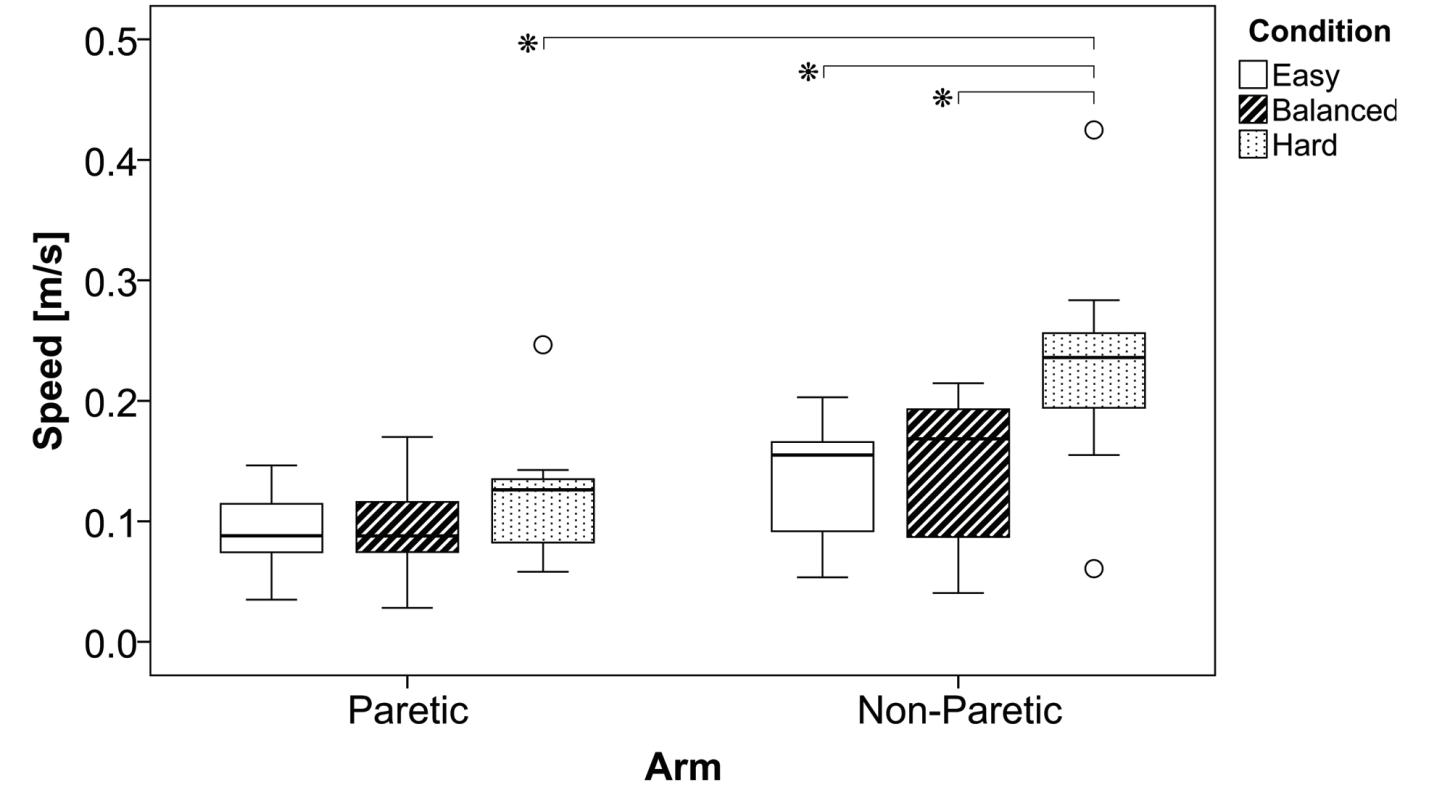
**Movement Speed**. Boxplots illustrate the movement speeds in m/s of the three conditions easy, balanced and hard for the paretic and non-paretic arm.

Reducing the available time to reach the target results in faster movement speeds (Figure 6). The ANOVA revealed a significant difference between the paretic and non-paretic arm (*F_1;59 _*= 19.346; *p *< 0.01; *η^2 ^*= 0.267), and showed a significant difference between the conditions (*F_2;59 _*= 6.013; *p *< 0.01; *η^2 ^*= 0.185), with no significant interactions between arm and conditions (*F_2;59 _*= 1.572; *p *= 0.217; *η^2 ^*= 0.056). Post hoc analysis revealed a significant difference between the paretic and non-paretic arm for the hard condition (p < 0.01). Significant differences were also found between the easy and hard conditions (p = 0.016) and the balanced and hard conditions (p = 0.033) in the non-paretic arm.

### Hand-Closing Time

**Figure 7:**
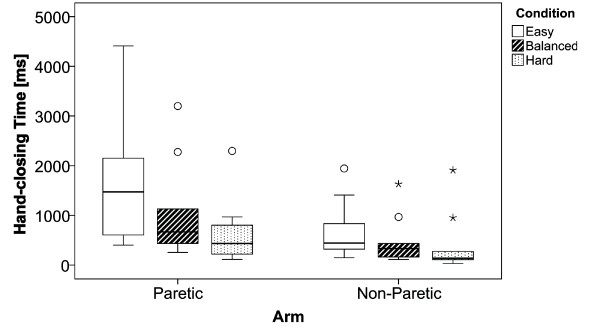
**Hand-Closing Time**. Boxplots showing the hand-closing time for the three different conditions easy, balanced and hard.

In contrast to the movement speed, the hand-closing time decreases with decreasing available time (Figure 7). While the ANOVA showed a significant difference between the paretic and non-paretic arm (*F_1;59 _*= 7.672; *p *< 0.01; *η^2 ^*= 0.126), no significant differences were found between the different conditions (*F_2;59 _*= 2.620; *p *= 0.082; *η^2 ^*= 0.09), and the interaction between arm and conditions (*F_2;59 _*= 0.806; *p *= 0.452; *η^2 ^*= 0.03).

### Reaction Time

**Figure 8:**
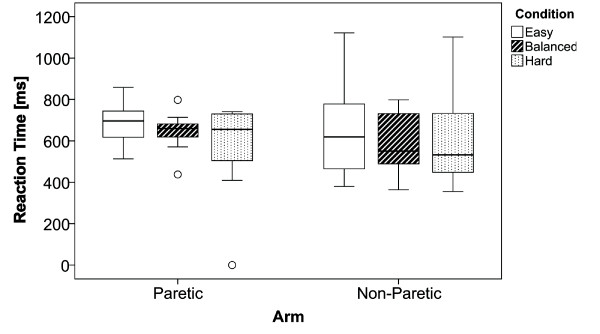
**Reaction Times**. Boxplots illustrating the reaction times.

Reaction time remained constant over the different conditions (Figure 8). The ANOVA did not show any significant difference between the paretic and non-paretic arm (*F_1;59 _*= 0.027; *p *= 0.871; *η^2 ^*= 0.001), no significant differences between the different conditions (*F_2;59 _*= 0.978; *p *= 0.383; *η^2 ^*= 0.036), and no significant differences for the interaction between arm and conditions (*F_2;59 _*= 0.423; *p *= 0.657; *η^2 ^*= 0.016).

### Hand-Path Ratio

**Figure 9:**
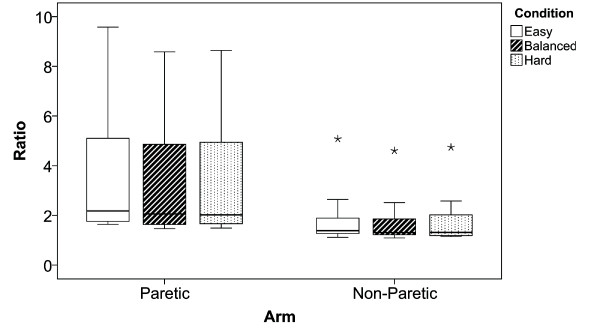
**Hand-Path Ratios**. Boxplots illustrating the hand-path ratios for the paretic and non-paretic arm.

Figure 9 illustrates the hand-path ratio for the paretic and non-paretic arm respectively. The ANOVA showed a significant difference between the paretic and non-paretic arm (*F_1;59 _*= 9.357; *p *< 0.01; *η^2 ^*= 0.150), no significant differences were found between the different conditions (*F_2;59 _*= 0.054; *p *= 0.947; *η^2 ^*= 0.002), and the interaction between arm and conditions (*F_2;59 _*= 0.014; *p *= 0.986; *η^2 ^*= 0.001).

### NASA-TLX

**Figure 10:**
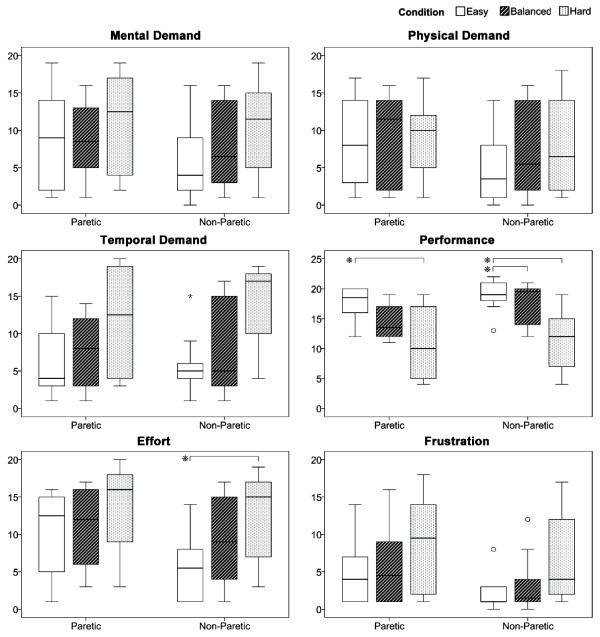
**NASA-TLX**. Boxplots illustrating the sub-scales of the NASA-TLX questionnaire for the paretic and non-paretic arms.

Figure 10 shows the mental, physical and temporal demand as well as the performance, effort and frustration sub-scales of the NASA-TLX. The ANOVA did not reveal any significant differences between the paretic and non-paretic arm for any of the sub-scales. Significant differences between conditions were found for the performance sub-scale in the paretic arm between the easy and hard condition (*p *< 0.01), in the non-paretic arm between the easy and hard conditions (*p *< 0.01) as well as in the non-paretic arm between the balanced and hard (*p *< 0.01) conditions. In the effort sub-scale a significant difference was found in the non-paretic arm for the easy and hard conditions (*p *= 0.04).

## Discussion

In the current study we investigated the applicability and validity of a mechanism that should be useful for adapting the difficulty of the exercise to the capabilities of the patient for upper-extremity rehabilitation. This difficulty balancing mechanism is based on Fitts' Law.

Our results show a decreasing amount of successful trials from the easy to the hard conditions. This seems obvious as patients had less time to reach the target on time and complete the trial. Since the ANOVA did not reveal any significant differences between the paretic and non-paretic arm, we can state that Fitts' Law is a valid mechanism to balance the difficulty of an exercise. Hence, although patients are capable of moving their non-paretic arm more accurately and faster, the number of successful trials is similar compared to when they perform the movements with their paretic arm.

The developed application further allowed assessing different movement characteristics, i.e., movement speed, hand-closing time, reaction time and the hand-path ratio. Unlike for the reaction times all other measurements showed a significant difference between the paretic and non-paretic arm reflecting the impaired motor control. While this seems obvious, the interesting aspect is that movement speed increased and hand-closing time decreased with increasing condition difficulty while the hand-path ratio stayed the same. Although one could assume that an increase of the exercise speed could cause decreased movement accuracy, this was not the case. It rather influenced whether the target was reached or not. Increased speed could however be used to train the hand-closing functionality and movement speed of the paretic arm. A subsequent study should thus assess the additional impact that the balancing mechanism may have on upper-extremity motor recovery after stroke.

Different theories on motivation state that performance is influenced by the effort a person exhibits during an exercise and the outcome they perceive. The effort, performance and frustration sub-scales of the NASA-TLX visualize this. Although the only significant difference for the paretic arm was found in the performance subscale, the others show a trend that, while patients increase their effort with increasing condition difficulty, they perceive their performance as getting worse and hence get frustrated. This eventually would lead to disengagement. Assuming that a success rate of 100% during an exercise my cause boredom and anything below 50% frustration, using Fitts' Law gives an initially challenging but at the same time not frustrating exercise difficulty. By manipulating the estimated time one can adjust the difficulty and thus influence a patients awareness of their capabilities, thereby increasing engagement during therapy.

Despite these findings, some limitations of the current study design need to be noted. In the current setup patients had to complete a trial by closing their hand when they reached the target. While this was useful to gain some insight into patients' hand-closing functionality, it at the same time limited the number of patients that were able to participate. Therefore, in a subsequent study, trial completion could be realized by asking patients to simply move over the target. This would allow inclusion of more patients with the drawback of losing one of the metrics.

In order to acquire the slopes and intercepts for the balancing mechanism, 50 trials were measured during the "assessment phase". As the results of Fitts' Law show, the correlations of the linear regressions are sometimes quite low. This probably caused sporadic inaccurate estimates during the different conditions. According to Figure 5, these, however, did not have a large effect on the number of successful trials. The number of assessment trials was limited to 50 because we did not want patients to get bored by the rather simple task. A subsequent study should assess the minimal amount of trials that are needed in order to obtain sufficiently high correlations.

Another interesting finding when looking at the correlations of the Fitts' Law data is the difference between the paretic and non-paretic arm. While patients 1, 2, 3, 9 and 10 had high correlations (R^2 ^> 0.7) for the paretic arm, they showed low correlations for the non-paretic arm. The opposite was found for patients 4, 5, 6, 7, 8 and 10. Although there seems to be a visible pattern when looking at this distribution, the clinical evaluation does not show any functional differences between the two groups that would allow drawing any conclusions. We rather suppose that the sometimes-low correlations for the non-paretic arm where due to the fact, that patients were not used to train using it. Subsequent studies should however further address this finding.

During the "exercise phase" each condition (easy/balanced/hard) was presented and performance measured for 2 minutes. Therefore the number of trials in each condition differed. This approach, compared to having a fixed number of trials per condition, was chosen to guarantee equal temporal lengths, preventing exhaustion, which could have negatively affected the performance during subsequent conditions.

In order to obtain a subjective measure of condition difficulty, patients were asked to give feedback using the NASA-TLX. Although nearly no significant differences were found, results show a trend indicating that condition difficulty is followed by an increase of mental demand, effort and frustration levels as well as by a decrease of performance. As we, however, also conclude on the motivation level of the patients, using a more established and motivation-oriented questionnaire, e.g., the intrinsic motivation inventory (IMI) [47] in subsequent studies would be of high importance. In the current study we chose the NASA-TLX over the IMI because of the smaller number of questions patients had to answer. This kept the cognitive demand low during the rest period.

The graphical implementation of the current application was kept quite simple, because the goal of the study was to verify the applicability of the balancing mechanism. We believe that, because of its simplicity, the mechanism can also be adapted to applications with higher graphical details.

## Conclusions

We developed an augmented feedback application with a balancing mechanism based on Fitts' Law and validated its applicability through a study with ten stroke patients. We were able to show that Fitts' Law indeed constitutes a powerful mechanism for task difficulty adaptation. Because it is physiologically valid and a widely used descriptor of reaching movements, we believe that Fitts' Law is an ideal estimator for upper-extremity rehabilitation in which patients have to relearn both speed and accuracy of movements. The simplicity of the mechanism further allows incorporating it in a large number of existing and novel augmented feedback applications, independent of the graphical settings those applications exhibit. Since studies have already shown that people only engage in an activity if the outcome matches the effort at which they perform [25-27], we encourage developers to incorporate such balancing mechanisms into their upcoming augmented feedback applications for rehabilitation. This may not only improve the effort that patients exhibit during therapy, but also increase their engagement and motivation over a longer period of time.

## Competing interests

LZ and LL are both employed by Hocoma AG, Volketswil, Switzerland, the producer of the Armeo^®^Spring. CK, RG, FM and RR declare to have no competing interests.

## Authors' contributions

LZ was involved in developing the software and the study design, analyzing data and drafting the manuscript. CK recruited subjects, acquired data, assisted with data analysis and revised the manuscript. LL and RG assisted with data interpretation and revised the manuscript. RR contributed in the formulation of the overall research questions and assisted in revising the manuscript. FM assisted in revising the manuscript. All authors read and approved the final manuscript.
